# Cholera Infantum

**Published:** 1876-06

**Authors:** 


*CHOLERA INFANTUM.*

In a few days this terrible disease will commence its summer campaign. If parents would provide themselves with "Van Deren's Remedy," prepared by Hall & Ruckle, 218 Greenwich Street, New York, they could completely control all bowel troubles and protect their children from cruel suffering, and perhaps death, which is too frequently their only relief.

